# Appropriateness of antibiotic prescribing in dental practice: results of two clinical audits conducted at a university hospital

**DOI:** 10.3389/froh.2026.1913184

**Published:** 2026-09-18

**Authors:** Romain Lan, Célia Lento, Delphine Tardivo, Carole Siani, Thomas Giraud, Jean-Hugues Catherine, Florence Carrouel, Nadim Cassir, Manon Roche

**Affiliations:** 1 Aix-Marseille University, AP-HM, CNRS, EFS, ADES, Timone Hospital, Department of Odontology, Marseille, France; 2 AP-HM, Service Central des Opérations Pharmaceutiques (SCOP), Marseille, France; 3 Aix-Marseille University, UMR 1252 SESSTIM (AMU, INSERM, IRD), Marseille, France; 4 Aix-Marseille University, CNRS, ISM, Marseille, France; 5 AP-HM, Hôpital Timone, Pôle PROMOD Odonto, Service Odontologie Hospitalière et Chirurgie Orale, Marseille, France; 6 Health Systemic Process (P2S), UR4129, University Claude Bernard Lyon 1, Lyon, France; 7 IHU Méditerranée Infection, Marseille, France; 8 Aix-Marseille Université, IRD, MEPHI, Marseille, France; 9 AP-HM, Marseille, France; 10 Aix-Marseille University, UMR CNRS 7273, Marseille, France

**Keywords:** antibiotic prescribing, antibiotic stewardship, antimicrobial resistance, clinical audit, dental infections, endodontics

## Abstract

Antimicrobial resistance (AMR) is a major global public health threat, and dentistry contributes substantially to unnecessary antibiotic use. Despite the publication of clear prescribing recommendations by the European Society of Endodontology (ESE) in 2018, inappropriate antibiotic prescribing remains common, particularly in dental emergency settings. This study aimed to evaluate the appropriateness of antibiotic prescribing in a university dental emergency department and to assess the impact of an audit-and-feedback intervention. A prospective two-phase clinical audit was conducted in 2025 at the University Hospital of Marseille. Two consecutive two-week audit periods were conducted before and after individualized feedback and educational workshops. Prescriptions were classified according to ESE guidelines as appropriate (A1, A2) or inappropriate (I1, I2). A secondary analysis evaluated the appropriateness of treatment modalities (A1C, A1NC). Statistical analyses were performed using Chi-squared or Fisher's exact tests. A total of 640 patients were included. Antibiotics were prescribed in 30.3% of consultations. The proportion of appropriate prescriptions did not improve significantly between audits (A1 + A2: 71.9% vs. 77.6%, *p* = 0.822). Among the 193 patients who received antibiotics, inappropriate prescriptions remained common (I2, 72.5%), particularly among patients presenting with pulpal or periapical diseases and among those consulting primarily because of pain. Among patients with a pulpal or periapical diagnosis who received antibiotics, 112 of 113 prescriptions (99.1%) were inappropriate, compared with 28 of 80 (35.0%) among other diagnoses. This pattern was consistent across the individual pulpal and periapical diagnostic subgroups. Inappropriate antibiotic prescribing remains frequent in dental emergency care. A single audit-and-feedback intervention was insufficient to significantly modify prescribing practices, highlighting the need for sustained and multifaceted antimicrobial stewardship strategies focused on improving diagnostic accuracy and ensuring timely operative management.

## 1 Introduction

In 2022, the World Health Organization's Global Antimicrobial Resistance and Use Surveillance System (GLASS) highlighted high levels of antimicrobial resistance (AMR) in 87 countries and urged actions to improve practices (1). The high morbidity and mortality associated with AMR make it a major public health challenge, and AMR is projected to become one of the leading causes of death worldwide by 2050. The burden of AMR has increased over recent decades, largely because of inappropriate antibiotic use, resulting in substantial economic costs (2), prolonged hospital stays, more expensive treatments (3), and increased mortality (4, 5). Therefore, there is an urgent need to promote the rational use of antibiotics across all healthcare disciplines, including dentistry, where inappropriate prescribing remains common despite the availability of clinical guidelines (6). In dental practice, antibiotics are prescribed primarily for the prevention and treatment of odontogenic infections, particularly those involving pulpal and periapical diseases (7–9). However, numerous international studies have shown that many antibiotic prescriptions issued by dentists are unnecessary or inappropriate and result from inaccurate diagnoses or poor adherence to clinical guidelines (10–14). Despite international efforts to reduce antibiotic prescribing, their overuse in dental practice remains widespread and continues to contribute substantially to antimicrobial resistance (15, 16). Common examples of misuse include prescribing antibiotics for dental pain in the absence of systemic signs of infection and inappropriate use for prophylaxis (17). To address this issue, the European Society of Endodontology (ESE) launched a campaign in 2018 promoting the appropriate use of antibiotics in light of the growing threat of AMR and the complexity of dental infections (18). The ESE subsequently published detailed recommendations for the management of endodontic diseases, including pulpal and periapical conditions, emphasizing the importance of accurate diagnosis and judicious antibiotic prescribing. According to these guidelines, antibiotics should be prescribed only in clearly defined clinical situations, primarily for acute odontogenic infections in medically compromised patients or in the presence of systemic involvement or progressive spread of infection in otherwise healthy individuals (19).

Furthermore, these recommendations emphasize that, whenever indicated, systemic antibiotics should be considered only as an adjunct to definitive local treatment rather than a substitute for operative management, except in situations requiring prophylactic therapy.

Similarly, the International Association of Dental Traumatology (IADT) does not recommend the routine use of systemic antibiotics for most traumatic injuries to permanent teeth, except in cases of tooth avulsion. However, antibiotic therapy may be justified depending on the patient's medical condition or the severity of the associated soft-tissue injury and its management (20, 21). Despite these recommendations, inappropriate antibiotic prescribing remains common in dentistry because of several factors, including diagnostic uncertainty—particularly in inflammatory conditions with an infectious component—patient expectations, and entrenched prescribing habits (13–15). Furthermore, the 2023 ESE S3-level Clinical Practice Guideline for the management of pulpal and periapical diseases provides an updated evidence-based framework for the treatment of these conditions, reaffirming that appropriate endodontic procedures, rather than systemic antibiotics, should constitute the primary therapeutic approach (22–26).

Given the complexity of odontogenic infections and the significant consequences of inappropriate antibiotic use, there is a clear need to evaluate the appropriateness of antibiotic prescribing practices in dental care. Accordingly, this study addressed two objectives. First, to evaluate whether an audit-and-feedback (A&F) intervention combined with educational sessions improved adherence to ESE recommendations, comparing antibiotic prescribing appropriateness before (Audit 1) and after (Audit 2) the intervention. Second, to describe the clinical situations in which inappropriate antibiotic prescribing was most frequently observed among patients who received antibiotics, with particular attention to diagnostic categories and reasons for prescribing. Particular attention was paid to the management of pulpal and periapical diseases because of their pathophysiological complexity and the diagnostic uncertainty frequently associated with these conditions. Beyond confirming that inappropriate prescribing is frequent, the specific contribution of this work is to combine a prospective two-phase design, a formal audit-and-feedback intervention with educational workshops, appropriateness assessment anchored on ESE stewardship recommendations, and a detailed characterization of the clinical situations in which inappropriate prescribing persisted after the intervention.

## 2 Materials and methods

### 2.1 Study design and setting

A prospective, two-phase clinical audit of antibiotic prescribing practices was conducted in 2025 in the dental emergency department of Marseille University Hospital, France. Two distinct two-week audit periods were conducted: April 7–18 (Audit 1) and October 13–24 (Audit 2). The study was conducted in accordance with the SQUIRE reporting guidelines for quality improvement research (Supplementary Table S1).

### 2.2 Audit and feedback intervention

The audit-and-feedback (A&F) intervention was designed according to the recommendations from the Joint Programming Initiative on Antimicrobial Resistance: Primary Care Antibiotic Audit and Feedback Network (JPIAMR-PAAN), adapted to the dental setting (27). Audit criteria were derived from the ESE guidelines (26) and focused first on the appropriateness of the indication for antibiotic prescribing and subsequently on the appropriateness of treatment modalities (choice of antibiotic, dosage, and treatment duration). Audit 1 served as the baseline evaluation. Data were collected prospectively during routine clinical practice using a standardized form integrated into the hospital information system (AXIGATE®) and anonymized prior to analysis. Results were first communicated to prescribers through written summary feedback comparing individual practices with guideline standards and benchmark data from peers. This was followed by two group sessions designed to foster discussion, peer learning, and clarification of clinical decision-making. Three months later, two educational workshops were conducted to address observed gaps identified in Audit 1, including the distribution of updated guidelines and decision-support tools.

Written individualized feedback reports were disseminated to all prescribers of the dental emergency department and included each practitioner's prescribing indicators (proportion of appropriate indications and compliance of molecule, dosage, and duration), benchmarked against ESE standards and anonymized peer performance. Each group feedback session lasted approximately 45 min, and each educational workshop lasted approximately 60 min; workshops were led by a senior dentist and a senior clinical pharmacist. All 13 practitioners working in the department during the study period (13/13; 100%) attended at least one feedback or educational session.

Audit 2 was performed six months after Audit 1, using the same methodology. The results of both audits were presented in aggregate form to the clinical teams, with individual data kept confidential. Measures were implemented to ensure secure handling of anonymized data and to facilitate the long-term integration of feedback into daily practice (Figure 1).

**Figure 1:**
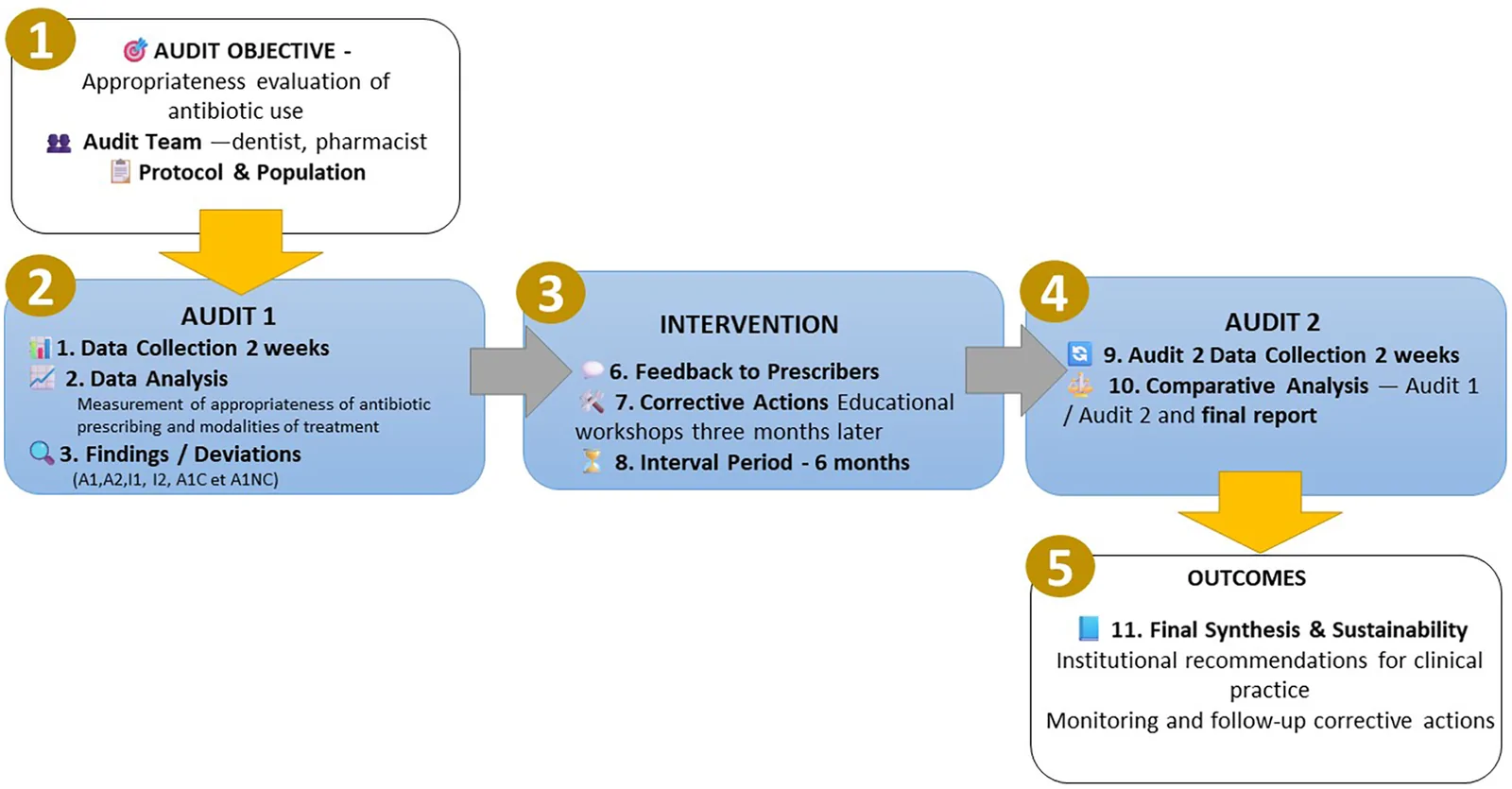
Antibiotic prescribing audit in dental emergency care—audit process flow chart.

### 2.3 Outcomes

The primary objective was to assess the appropriateness of antibiotic prescribing and, when antibiotics were indicated, the appropriateness of treatment modalities in the dental emergency department, according to the ESE guidelines, before and after the implementation of the audit-and-feedback intervention and educational sessions. The secondary objective was to describe the clinical situations in which inappropriate antibiotic prescribing was most frequently observed among patients who received antibiotics, with particular attention to diagnostic categories and reasons for prescribing.

The appropriateness of antibiotic prescribing was assessed in two sequential steps. First, the indication for antibiotic therapy was evaluated according to the clinical presentation and ESE recommendations. Prescriptions were classified into four categories: A1, antibiotic indicated and prescribed (appropriate prescription); A2, antibiotic not indicated and not prescribed (appropriate non-prescription); I1, antibiotic indicated but not prescribed (inappropriate under-prescription); I2, antibiotic not indicated but prescribed (inappropriate over-prescription).

The correspondence between each clinical diagnosis and the ESE-based indication used for this classification is detailed in Supplementary Table S2, which maps every diagnostic category to the applicable ESE recommendation to ensure transparency and reproducibility of the appropriateness assessment. This classification framework was drawn up before the first audit and was reviewed and agreed jointly by a senior clinical pharmacist and a senior dentist, who resolved uncertain scenarios by consensus against the framework rather than by independent duplicate rating; formal inter-rater agreement statistics were therefore not computed.

In a second step, only prescriptions classified as A1 (appropriate indication) were further evaluated for compliance with the recommended treatment modalities (choice of antibiotic, dosage, route of administration, dosing frequency, and treatment duration). These prescriptions were classified as follows: A1C, fully compliant with ESE recommendations (appropriate indication and compliant treatment modality); A1NC, appropriate indication but non-compliant treatment modality.

Cases classified as I1 (indicated but not prescribed) were excluded from the second-step analysis because treatment modalities could not be assessed. Furthermore, in accordance with the recommendations of the JPIAMR-PAAN (27), the audit focused primarily on antibiotic misuse. Accordingly, antibiotic misuse was defined as either inappropriate over-prescribing (I2) or non-compliance with the recommended treatment modalities despite an appropriate indication (A1NC). The classification process is illustrated in Figure 2. I1 cases were nevertheless retained in the primary Step-1 comparison between audits and are described in full in Supplementary Table S3, so that under-prescribing remains visible alongside over-prescribing.

**Figure 2:**
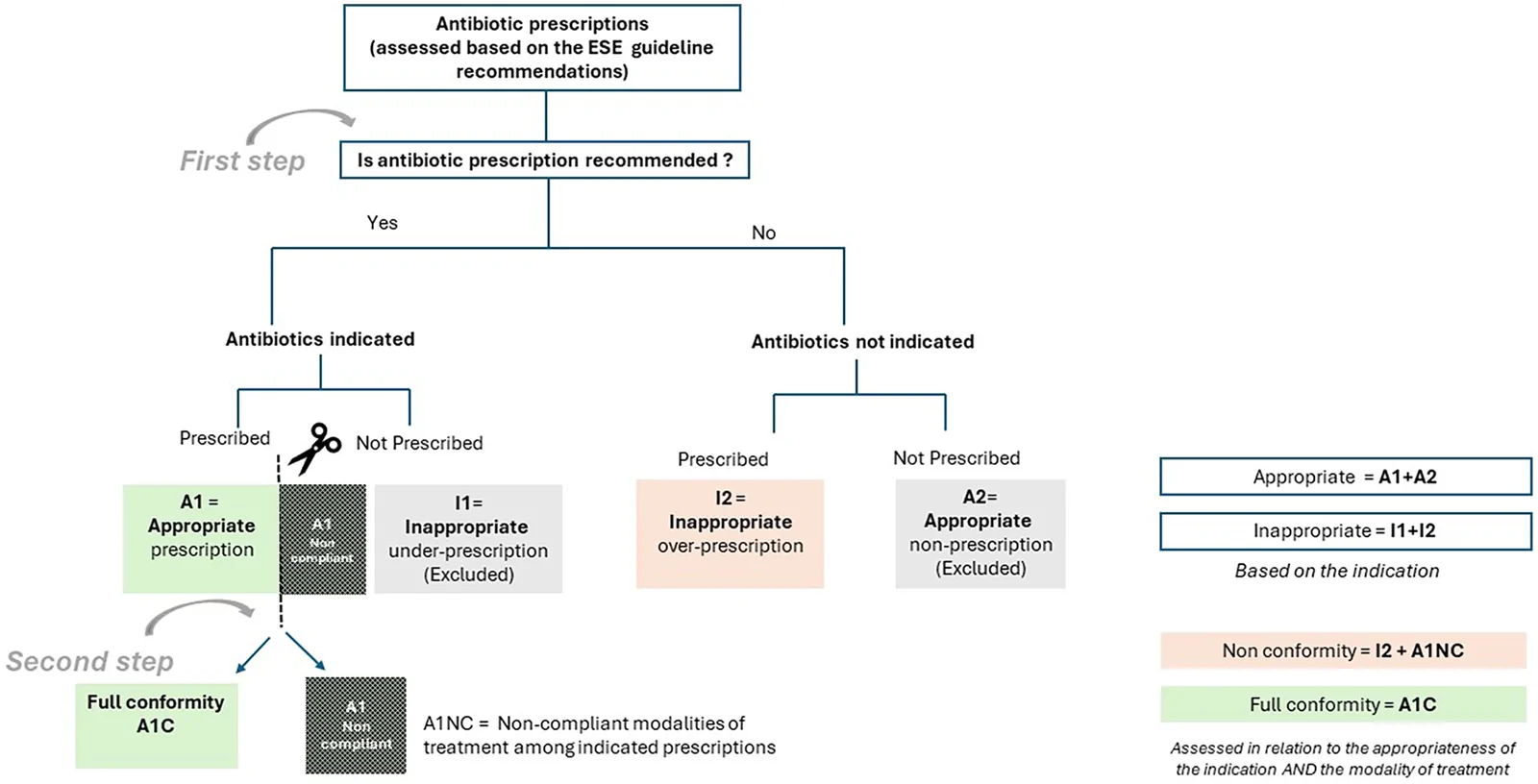
Schematic representation of antibiotic prescription classification. Prescriptions were categorized according to ESE guideline recommendations and prescribing behavior. Cases of indicated but non-prescribed antibiotics (I1) were excluded from the analysis. Antibiotic misuse comprised inappropriate treatment modalities among indicated cases (A1NC) and inappropriate over-prescription (I2).

### 2.4 Study population

Patients attending the dental emergency department during either audit period were eligible for inclusion. Patients with incomplete medical records were excluded. Immune status was classified into two categories: (i) the general population and (ii) high-risk patients, including individuals with immunosuppression or risk factors for infective endocarditis, according to the 2023 European Society of Cardiology (ESC) guidelines (28).

### 2.5 Sample size

The sample size was calculated using the formula for comparing two independent proportions. Based on institutional experience and published estimates of baseline appropriateness of antibiotic prescribing in dental practice (typically ranging from 50% to 80% across different settings), the baseline appropriateness rate was assumed to be 70% (29–31). An absolute improvement of 10 percentage points (from 70% to 80%) following the audit-and-feedback intervention was considered clinically meaningful.

This assumption was supported by previous studies demonstrating that audit-and-feedback and antimicrobial stewardship interventions can reduce inappropriate antibiotic prescribing in dentistry, although the magnitude of the effect varies according to the intervention and clinical setting. For example, Elouafkaoui et al. reported a 5.7% reduction in overall antibiotic prescribing in Scotland following written individualized feedback (29), whereas Teoh et al. observed a 44.6% reduction in inappropriate prescriptions after an educational antimicrobial stewardship programme (30). Similarly, Mendez-Romero et al. reported reductions of up to 70% in non-compliant antibiotic prescriptions in Latin America (31). Based on these findings, an expected improvement of 8–10 percentage points was considered both conservative and realistic.

Assuming a two-sided *α* level of 5% and a statistical power of 80%, the minimum required sample size was estimated at 199 patients per audit period. The achieved sample size was determined by the consecutive caseload of the dental emergency department of Timone Hospital during the two predefined audit periods; both periods exceeded the required number of patients.

Although the overall sample exceeded the planned size, the distribution of cases across categories was uneven. For descriptive analyses, diagnoses were therefore grouped into “pulpal and periapical diseases” and “other diagnoses”. The latter category includes clinically heterogeneous conditions, and this grouping is acknowledged as a limitation. The distribution across individual pulpal and periapical diagnoses is reported separately in Supplementary Table S4.

### 2.6 Data collection

Demographic variables (age and sex), clinical variables (immune status, reason for consultation, diagnosis, type of procedure performed, and reason for non-intervention), and therapeutic variables (antibiotic prescribed, dosage, dosing interval, treatment duration, and prior antibiotic exposure) were collected prospectively using a standardized data collection form validated by both a senior clinical pharmacist and a senior dentist. Age was categorized into three groups: children (0–16 years), adults (17–65 years), and older adults (>65 years).

### 2.7 Statistical analysis

Descriptive statistics were used to characterize the study population and antibiotic prescribing patterns. Categorical variables were summarized as frequencies and percentages. Comparisons between Audit 1 and Audit 2 were performed using the Chi-squared test or Fisher's exact test, as appropriate, depending on the expected cell counts. Differences in prescribing appropriateness before and after the intervention were assessed using the Chi-squared test for independent samples.

For the secondary objective, analyses were restricted to patients who received antibiotics and compared appropriate prescriptions (A1) with inappropriate over-prescriptions (I2). Categorical variables were summarized as frequencies and row percentages, and comparisons between clinical categories were performed using the Chi-squared test or Fisher's exact test, as appropriate. Because of small cell counts within individual diagnostic categories, these analyses were considered descriptive and no multivariable modelling was performed.

All statistical analyses were performed using Python 3.11.7 (Python Software Foundation) with the pandas 2.1.4, NumPy 1.26.3, statsmodels 0.14.1, scikit-learn 1.4.0 and Matplotlib 3.8.2 libraries. Records with incomplete baseline information were excluded before statistical analysis, as detailed in the Results; consequently, no missing data remained in the final analytical dataset for either the primary or secondary outcomes. Statistical significance was defined as *p* < 0.05.

## 3 Results

### 3.1 Population characteristics and general clinical data

A total of 668 patients were screened across the two audit periods. After excluding four patients from Audit 1 and 24 from Audit 2 because of incomplete data, the final study population comprised 640 patients: 377 in Audit 1 and 263 in Audit 2 (Figure 3).

**Figure 3:**
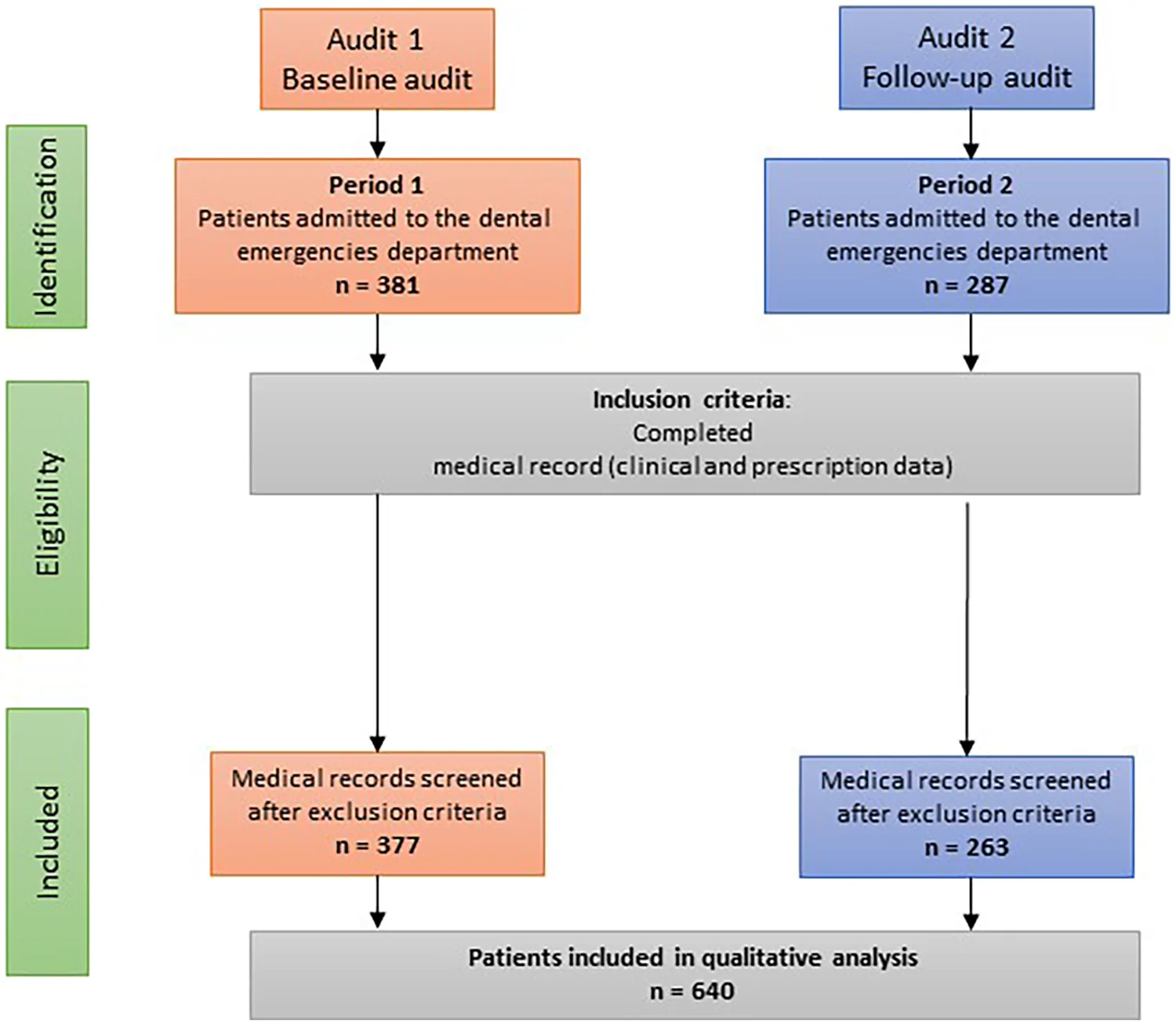
Screening flowchart.

Patient demographics and clinical characteristics are presented in Supplementary Table S5. The two cohorts were comparable with regard to sociodemographic characteristics, including age group and immune status, although a significant difference was observed in gender distribution (*p* = 0.037). Clinical characteristics, including the reason for consultation and diagnosis, were similar between the two audit periods.

In contrast, clinical management differed significantly between audits, with a change in the proportion of patients receiving a dental intervention versus those managed without intervention (*p* = 0.026), reflecting evolving clinical practice patterns.

In both audit periods, pain was the leading reason for consultation (78.5% in Audit 1 and 79.1% in Audit 2). Pulpal and periapical diseases were the most common diagnoses (60.2% and 60.5%, respectively). Dental interventions were performed in more than half of the patients, although their frequency decreased from 60.2% in Audit 1 to 51.3% in Audit 2.

### 3.2 Objective 1—effect of the audit-and-feedback intervention

#### 3.2.1 Descriptive analysis of antibiotic prescribing

Among the study population, antibiotics were prescribed to 124 patients in Audit 1 (32.9%) and 69 patients in Audit 2 (26.2%). Characteristics of patients who received an antibiotic prescription are summarized in Tables 1, 2.

**Table 1:** Patient and clinical characteristics among patients receiving antibiotic prescriptions, by audit period.

Characteristic	Audit 1 (*n* = 124)	Audit 2 (*n* = 69)	*p*-value
Age group, *n* (%)			0.350
0–16 years	20 (16.1%)	6 (8.7%)	
17–65 years	94 (75.8%)	57 (82.6%)	
>65 years	10 (8.1%)	6 (8.7%)	
Gender, *n* (%)			0.109
Male	68 (54.8%)	46 (66.7%)	
Female	56 (45.2%)	23 (33.3%)	
Immune status, *n* (%)			0.877
General population	105 (84.7%)	59 (85.5%)	
High-risk population	19 (15.3%)	10 (14.5%)	
Reason for consultation, *n* (%)			0.474
Pain	103 (83.1%)	60 (87.0%)	
Other^a^	21 (16.9%)	9 (13.0%)	
Diagnosis, *n* (%)			0.625
Pulpal and periapical diseases^b^	71 (57.3%)	42 (60.9%)	
Other diagnoses^c^	53 (42.7%)	27 (39.1%)	

a Other reasons for consultation: swelling, follow-up/control visits, prosthetic problems, dental trauma.

b Pulpal and periapical diseases: caries, acute infections, symptomatic pulpitis, pulpal diseases.

c Other diagnoses: periodontal diseases, dental trauma, other oral/dental infections (osteitis, cellulitis, eruption-related infections, infectious diseases of the oral mucosa), non-urgent dental problems.

**Table 2:** Characteristics of antibiotic prescriptions, by audit period.

Characteristic	Audit 1 (*n* = 124)	Audit 2 (*n* = 69)	*p*-value
Prescribed antibiotic molecule, *n* (%)			0.570
Amoxicillin only	85 (68.5%)	50 (72.4%)	
Other antibiotic regimens^a^	39 (31.5%)	19 (27.6%)	
Management strategy, *n* (%)			0.898
Dental intervention	42 (33.9%)	24 (34.8%)	
Antibiotic prescription without dental intervention	82 (66.1%)	45 (65.2%)	
Reasons for antibiotic prescription without dental intervention (Audit 1: *n* = 82; Audit 2: *n* = 45), *n* (%)			NA
Clinical situation	12 (14.6%)	11 (24.4%)	
Infection context	31 (37.8%)	22 (48.8%)	
Intervention difficulty	13 (15.8%)	4 (8.9%)	
Lack of organization	26 (31.7%)	8 (17.8%)	

a Other antibiotic regimens: amoxicillin/clavulanic acid, clindamycin, azithromycin, metronidazole, amoxicillin + metronidazole.

No significant differences were observed between the two audits regarding age, gender distribution, reasons for consultation, diagnostic categories, prescribed antibiotic agents, or management strategy (antibiotic prescription alone versus combined with a dental intervention).

Amoxicillin alone was the most frequently prescribed antibiotic in both audits (68.5% in Audit 1 vs. 72.4% in Audit 2, *p* = 0.570). Pain remained the predominant reason for consultation among patients receiving antibiotics (83.1% vs. 87.0%, *p* = 0.474), while pulpal and periapical diseases were the most frequent diagnoses associated with antibiotic prescriptions (57.3% vs. 60.9%, *p* = 0.625). Approximately two-thirds of antibiotic prescriptions were issued without a dental intervention in both audits (66.1% vs. 65.2%, *p* = 0.898).

#### 3.2.2 Appropriateness of antibiotic prescribing before and after the intervention

When considering the overall study population (Table 3), no significant differences were observed between the two audits in the appropriateness of antibiotic prescribing. Appropriate prescribing (A1 + A2) accounted for 71.9% of cases in Audit 1 and 77.6% in Audit 2, and inappropriate prescribing (I1 + I2) decreased from 28.1% to 22.4%; the overall comparison of appropriate versus inappropriate prescribing between audits was not statistically significant (*p* = 0.105).

**Table 3:** Appropriateness of antibiotic prescribing between Audit 1 and Audit 2. Panel A: appropriateness of the indication (Step 1). Panel B: appropriateness of treatment modalities among patients receiving antibiotics (Step 2).

Panel A—Step 1: appropriateness of the antibiotic indication (overall population, *N* = 640)			
Prescribing appropriateness	Audit 1 (*n* = 377)	Audit 2 (*n* = 263)	*p*-value
Inappropriate prescribing, *n* (%)	106 (28.1%)	59 (22.4%)	0.166
Antibiotics indicated but not prescribed (I1)	13 (12.3%)	12 (20.3%)	
Antibiotics not indicated but prescribed (I2)	93 (87.7%)	47 (79.7%)	
Appropriate prescribing, *n* (%)	271 (71.9%)	204 (77.6%)	0.822
Antibiotics indicated and prescribed (A1)	31 (11.4%)	22 (10.8%)	
Antibiotics not indicated and not prescribed (A2)	240 (88.6%)	182 (89.2%)	
Comparison between inappropriate and appropriate prescribing			0.105

Percentages for I1/I2 and A1/A2 are calculated within the corresponding appropriateness category.

**Table:** 

Panel B—Step 2: appropriateness of treatment modalities (patients receiving antibiotics, *n* = 193)			
Treatment-modality appropriateness	Audit 1 (*n* = 124)	Audit 2 (*n* = 69)	*p*-value
Non-conformity with prescribing recommendations, *n* (%)	106 (85.5%)	57 (82.6%)	0.356
Antibiotics not indicated but prescribed (I2)	93 (75.0%)	47 (68.1%)	
Non-compliant modalities among indicated prescriptions (A1NC)	13 (10.5%)	10 (14.5%)	
Full conformity: appropriate indication and compliant prescription (A1C), *n* (%)	18 (14.5%)	12 (17.4%)	0.750
Comparison between non-conformity and full conformity			0.597

Treatment-modality appropriateness was assessed among indicated prescriptions according to guideline recommendations.

Among patients who received an antibiotic prescription (Table 3), the distribution of prescribing conformity categories was also comparable between the two audit periods. Overall, non-conformity with prescribing guidelines accounted for 85.5% of prescriptions in Audit 1 and 82.6% in Audit 2 (*p* = 0.356). Most of these non-conformities corresponded to prescriptions issued without a clinical indication (I2), representing 75.0% and 68.1% of prescriptions in Audits 1 and 2, respectively. Prescriptions that were clinically indicated but did not comply with treatment recommendations (A1NC) were less frequent (10.5% and 14.5%, respectively).

Fully compliant prescriptions—that is, prescriptions that were both clinically indicated and correctly issued (A1C)—represented only 14.5% of antibiotic prescriptions in Audit 1 and 17.4% in Audit 2, with no significant difference between audits (*p* = 0.750).

Similarly, the comparison between appropriate and inappropriate prescribing showed no significant differences between the two audit periods, either in the overall study population (*p* = 0.105) or among patients who received antibiotics (*p* = 0.597). Under-prescribing (I1) was infrequent in both periods (13/377 and 12/263 patients); the distribution of all four appropriateness categories is reported in Supplementary Table S6.

### 3.3 Objective 2—clinical situations associated with inappropriate antibiotic prescribing

To investigate the prescribing decision itself, a focused analysis was performed among patients who received antibiotics, comparing appropriate prescriptions (A1) with inappropriate overprescribing in the absence of a clinical indication (I2). Of the 193 patients who received an antibiotic across both audits, 27.5% were classified as having received an appropriate prescription (A1), whereas 72.5% received an inappropriate prescription (I2) (Table 4).

**Table 4:** Comparison of appropriate (A1) and inappropriate (I2) prescriptions among the 193 patients who received antibiotics. Percentages are calculated by row (each predictor category sums to 100% across A1 and I2).

Characteristic	A1, *n* (%)	I2, *n* (%)	Total	*p*-value
Reason for consultation				<0.001
Pain	36 (22.1%)	127 (77.9%)	163	
Other^a^	17 (56.7%)	13 (43.3%)	30	
Diagnosis				<0.001
Pulpal and periapical diseases^b^	1 (0.9%)	112 (99.1%)	113	
Other diagnoses^c^	52 (65.0%)	28 (35.0%)	80	
Management strategy				0.037
Dental intervention	12 (18.2%)	54 (81.8%)	66	
Antibiotic without dental intervention	41 (32.3%)	86 (67.7%)	127	
Reason for antibiotic without dental intervention (A1: *n* = 41; I2: *n* = 86)				—
Clinical situation	0 (0.0%)	23 (100%)	23	
Infectious context	39 (73.6%)	14 (26.4%)	53	
Difficulty performing intervention	1 (5.9%)	16 (94.1%)	17	
Organizational constraints	1 (2.9%)	33 (97.1%)	34	

a,b,c See footnotes to Table 1. A per-audit breakdown (Audit 1 vs. Audit 2) is provided in Supplementary Table S3.

Presented with percentages calculated within each clinical category (row percentages), the associations were as follows. Pain was more frequently the reason for consultation among inappropriate prescriptions: among patients consulting for pain, 22.1% received an appropriate prescription and 77.9% an inappropriate one, whereas among other consultation reasons 56.7% were appropriate. Diagnoses differed markedly: among patients with a pulpal or periapical diagnosis, 99.1% of prescriptions were inappropriate, compared with 35.0% among other diagnoses. Regarding management, antibiotics issued without a dental intervention were appropriate in 32.3% of cases versus 18.2% when a dental procedure was performed. Among patients managed without a dental intervention, an infectious context was associated with appropriate prescribing (73.6% appropriate), whereas an ill-defined clinical situation or organizational constraints were almost exclusively associated with inappropriate prescribing (100% and 97.1% inappropriate, respectively). All bivariate comparisons were statistically significant (*p* < 0.05).

Within the “pulpal and periapical diseases” category, the pattern of inappropriate prescribing was uniform across the individual diagnoses (Supplementary Table S4). Inappropriate prescriptions accounted for all prescriptions issued for deep caries (12/12), symptomatic pulpitis (23/23) and other pulpal diseases (7/7), and for 70 of the 71 prescriptions issued for acute apical infections. The single appropriate prescription recorded in this category corresponded to an acute apical infection, which is the only pulpal or periapical condition for which the ESE guidelines recognise an indication, and then only in the presence of systemic involvement or spreading infection. Acute apical infections also represented the majority of the category (71/113; 62.8%), indicating that most inappropriate prescribing followed the diagnosis of a localised acute infection that should have been managed by drainage or endodontic treatment alone.

## 4 Discussion

This two-phase clinical audit provides an updated assessment of antibiotic prescribing practices in a university dental emergency department and highlights persistent gaps in adherence to evidence-based prescribing guidelines. Despite the implementation of an audit-and-feedback intervention combined with educational sessions, no significant improvement in prescribing appropriateness was observed between the two audit periods. In both phases, inappropriate prescriptions—mainly antibiotics prescribed without a clinical indication (I2)—accounted for more than two-thirds of antibiotic use, underscoring the persistent challenge of reducing unnecessary antibiotic prescribing in dental emergency settings.

Pain was the predominant reason for consultation and was frequently observed among patients receiving with inappropriate antibiotic prescriptions, consistent with previous studies identifying pain-related diagnostic uncertainty as a major contributor to unnecessary antibiotic use. Pulpal and periapical diseases represented the diagnostic category most frequently associated with inappropriate prescribing, whereas appropriate prescriptions were mainly observed in other diagnostic situations or more complex infectious presentations. These findings are in line with the recommendations of the ESE guidelines (2018) and the 2023 S3-level Clinical Practice Guideline, which emphasize that pulp-related conditions should primarily be managed through operative treatment rather than systemic antibiotic therapy (19, 26).

The audit-and-feedback intervention did not result in a measurable improvement in antibiotic prescribing appropriateness. Although a non-significant positive trend was observed, several factors may explain the limited impact of the intervention. Dental emergency departments are characterized by high patient volumes, time constraints, and limited availability of immediate operative care, which may encourage clinicians to prescribe antibiotics as a temporary management strategy (32, 33). In addition, patient expectations and misconceptions regarding the role of antibiotics in the management of dental pain may contribute to continued overprescribing. It should also be noted that the overall antibiotic prescribing rate fell from 32.9% (124/377) in Audit 1 to 26.2% (69/263) in Audit 2. Although this difference was not statistically significant, it may represent a clinically relevant signal that warrants evaluation in larger studies or repeated audit cycles.

In contrast, audit-and-feedback interventions have demonstrated reductions in inappropriate antibiotic prescribing in primary care settings (31–36), although their effectiveness depends on intervention intensity, repetition, and integration into practice. The intensity and single-cycle nature of our intervention, together with a six-month interval between audits, may have been insufficient to induce measurable behavioural change; repeated feedback cycles are generally required to achieve durable improvement. In addition, because the study was powered to detect a 10-percentage-point absolute improvement, it may have been underpowered to detect smaller but clinically meaningful changes. The observed absolute difference in appropriateness was of the order of 5.7 percentage points, i.e., approximately half the difference the study was designed to detect.

Previous studies have emphasized the potential benefit of providing individualized feedback to prescribers. Compared with group-level feedback, personalized feedback offers greater specificity, facilitates self-reflection, and may promote behavioural change. According to the Clinical Performance Feedback Intervention Theory (CP-FIT), active feedback delivery, peer discussion, and social interaction enhance the effectiveness of audit-and-feedback interventions by increasing engagement with performance assessment and supporting improvement strategies (27).

Our descriptive analysis identified a marked concentration of inappropriate prescribing among pulpal and periapical conditions. Among patients with these diagnoses who received antibiotics, 112 of 113 prescriptions (99.1%) were inappropriate, and this pattern was consistent across the individual diagnostic subgroups. These findings highlight pulpal and periapical conditions as an important target for future antimicrobial stewardship interventions. Improving diagnostic accuracy, particularly in distinguishing conditions requiring operative management from those in which systemic antibiotics are indicated, may therefore represent an important strategy for reducing unnecessary antibiotic use.

The high proportion of antibiotic prescriptions issued without a dental intervention—approximately two-thirds of cases in both audits—is noteworthy. This pattern may reflect organizational constraints such as emergency workload, limited appointment availability, treatment complexity, and reduced access to same-day operative care, as well as patient-related factors; our descriptive data showed that inappropriate prescriptions without intervention occurred mainly in the context of organizational constraints and ill-defined clinical situations. Previous studies have shown that when operative treatment cannot be performed, antibiotics are frequently used as a substitute, despite their inability to resolve pulpal pain or treat the underlying pathology (18, 37).

These findings are consistent with international evidence demonstrating widespread antibiotic misuse in endodontics and emergency dental care (37–43). Several studies have reported that a substantial proportion of antibiotics prescribed for dental conditions are unnecessary, particularly when driven by symptoms related to pulpitis and dental pain (37–43). The complex pathophysiology of pulpal and periapical diseases, in which inflammatory and microbial processes may coexist, contributes to diagnostic uncertainty and may increase the risk of inappropriate antibiotic prescribing (38, 43).

This study has several practical implications. First, improving diagnostic support through clinical decision-support tools, standardized triage algorithms, or rapid access to endodontic consultation may help reduce inappropriate prescribing. Second, repeated audit-and-feedback cycles combined with collective training and peer discussion may be necessary to achieve sustained behavioural change (44–46). Third, integrating infectious-disease expertise into dental emergency pathways, through specialist consultation or multidisciplinary stewardship initiatives, could further strengthen antibiotic management.

### 4.1 Limitations

This study was conducted in a single academic centre, which may limit generalizability. The interval between audit periods may have been insufficient to detect measurable behavioural change, and the study was powered only for a 10-percentage-point improvement. Prescribing appropriateness was assessed according to ESE guidelines, which provide a widely accepted framework but may not fully reflect local practice variations. Several additional limitations should be acknowledged. First, appropriateness was classified using a standardized ESE-based framework reviewed jointly by senior dentists and pharmacists before study initiation (Supplementary Table S2), but formal inter-rater agreement (e.g., Cohen's *κ*) was not computed. Second, the broad “pulpal and periapical diseases” category groups clinically distinct entities (symptomatic irreversible pulpitis, symptomatic apical periodontitis, acute apical abscess, chronic apical pathology). Although the distribution of inappropriate prescribing was consistent across the individual diagnoses reported, the small numbers within individual diagnostic subgroups limit more detailed comparisons, and future studies should analyse these conditions separately. The “other diagnoses” category is also clinically heterogeneous and includes conditions with differing thresholds for antibiotic indication. Third, individual prescribers were not identified in the dataset, which precluded personalized feedback trajectories and prescriber-level analysis. Finally, unmeasured factors—including prescriber experience, workflow constraints, and patient expectations—may have influenced prescribing decisions.

## 5 Conclusion

In this clinical audit, inappropriate antibiotic prescribing remained frequent in dental emergency care, with a particularly high proportion of inappropriate prescriptions observed among patients with pulpal and periapical diseases, where operative treatment should be prioritized and systemic antibiotics reserved for situations with a clear indication or used as an adjunct when necessary. A single audit-and-feedback cycle was insufficient to significantly improve prescribing practices, highlighting the need for sustained and multifaceted antimicrobial stewardship strategies. Improving diagnostic accuracy, facilitating access to operative care, and reinforcing adherence to evidence-based prescribing recommendations will be essential to reduce unnecessary antibiotic use and limit the contribution of dental care to antimicrobial resistance.

## Data Availability

The original contributions presented in the study are included in the article/Supplementary Material, further inquiries can be directed to the corresponding author.
