# Supplementary material for: Appropriateness of antibiotic prescribing in dental practice: results of two clinical audits conducted at a university hospital
Source: Front Oral Health. 2026 Sep 18;7:1913184. doi: 10.3389/froh.2026.1913184 (PMC13630595; doi:10.3389/froh.2026.1913184)
Supplement: Supplementary file 2 [file Table2.docx]

**Supplementary Table 2.** Mapping of clinical diagnoses to ESE-based antibiotic prescribing recommendations used for appropriateness classification (response to Reviewer 2).

| **Study category** | **Specific condition** | **Antibiotic indicated?** | **Recommended first-line management** | **Ref.** |
| --- | --- | --- | --- | --- |
| Pulpal & periapical diseases | Deep caries / reversible pulpitis (vital pulp) | No | Selective caries removal, pulp capping/restoration | 19, 27 |
|  | Symptomatic irreversible pulpitis | No | Pulpotomy / pulpectomy / RCT or extraction | 18, 27 |
|  | Pulp necrosis / symptomatic apical periodontitis | No | Chemo-mechanical debridement (RCT) or extraction | 18, 27 |
|  | Acute apical abscess — localised, no systemic signs | No | Incision & drainage / pulpectomy / extraction | 18, 27 |
|  | Acute apical abscess WITH systemic involvement, spreading infection, or immunocompromised host | **Yes (adjunct)** | Operative drainage PLUS systemic antibiotic | 18, 27 |
|  | Chronic apical abscess / sinus tract | No | RCT or extraction | 18, 27 |
| Other diagnoses | Gingivitis / chronic periodontitis | No | Mechanical debridement | 27 |
|  | Periodontal / pericoronal abscess — localised | No | Drainage/debridement; antibiotic only if systemic spread | 18, 27 |
|  | Necrotising periodontal disease | **Yes if systemic signs** | Debridement + systemic antibiotic | 27 |
|  | Cellulitis / spreading fascial-space infection | **Yes (adjunct)** | Urgent drainage PLUS systemic antibiotic | 18, 27 |
|  | Osteitis / osteomyelitis | **Yes** | Systemic antibiotic + surgical management | 18 |
|  | Dental trauma (except avulsion of permanent teeth) | No (routine) | Per IADT; antibiotic reserved for avulsion / severe soft-tissue injury | 20, 21 |
|  | Oral mucosal infectious disease | Aetiology-dependent | Aetiology-directed treatment | 18 |
|  | Non-urgent / prosthetic / follow-up | No | Local / conservative care | — |
|  | High-risk cardiac patient before invasive procedure | **Yes (prophylaxis)** | Antibiotic prophylaxis per ESC 2023 | 29 |

*Classification derived from ESE recommendations (18,19,27), IADT guidelines (20,21) and ESC 2023 (29 for prophylaxis), reviewed jointly by a senior dentist and a senior clinical pharmacist prior to study initiation. Antibiotics were considered indicated only in the presence of systemic involvement, spreading infection, or a documented prophylactic indication.*
