# Supplementary material for: Appropriateness of antibiotic prescribing in dental practice: results of two clinical audits conducted at a university hospital
Source: Front Oral Health. 2026 Sep 18;7:1913184. doi: 10.3389/froh.2026.1913184 (PMC13630595; doi:10.3389/froh.2026.1913184)
Supplement: Supplementary file 3 [file Table3.docx]

**Supplementary Table 3.** Distribution of the four prescribing appropriateness categories by audit period (response to Reviewer 1, comment 5).

| **Category** | **Audit 1 (n = 377)** | **Audit 2 (n = 263)** | **Total (N = 640)** |
| --- | --- | --- | --- |
| A1 — indicated & prescribed | 31 (8.2%) | 22 (8.4%) | 53 (8.3%) |
| A2 — not indicated & not prescribed | 240 (63.7%) | 182 (69.2%) | 422 (65.9%) |
| I1 — indicated but not prescribed | 13 (3.4%) | 12 (4.6%) | 25 (3.9%) |
| I2 — not indicated but prescribed | 93 (24.7%) | 47 (17.9%) | 140 (21.9%) |
