# Supplementary material for: Appropriateness of antibiotic prescribing in dental practice: results of two clinical audits conducted at a university hospital
Source: Front Oral Health. 2026 Sep 18;7:1913184. doi: 10.3389/froh.2026.1913184 (PMC13630595; doi:10.3389/froh.2026.1913184)
Supplement: Supplementary file 4 [file Table4.docx]

**Supplementary Table 4.** Distribution of appropriate (A1) and inappropriate (I2) prescriptions across the specific diagnoses grouped within the "pulpal and periapical diseases" category (n = 113; response to Reviewer 2).

| **Specific diagnosis** | **A1, n** | **I2, n** | **Total** | **% inappropriate** |
| --- | --- | --- | --- | --- |
| Deep caries | 0 | 12 | 12 | 100.0% |
| Symptomatic pulpitis | 0 | 23 | 23 | 100.0% |
| Acute infections (apical abscess) | 1 | 70 | 71 | 98.6% |
| Other pulpal diseases | 0 | 7 | 7 | 100.0% |
| **Total** | **1** | **112** | **113** | **99.1%** |

*Percentages are calculated by row. The single appropriate prescription recorded in this category corresponded to an acute apical infection, the one pulpal or periapical condition for which the ESE guidelines recognise an indication when systemic involvement or spreading infection is present.*
