# Supplementary material for: Appropriateness of antibiotic prescribing in dental practice: results of two clinical audits conducted at a university hospital
Source: Front Oral Health. 2026 Sep 18;7:1913184. doi: 10.3389/froh.2026.1913184 (PMC13630595; doi:10.3389/froh.2026.1913184)
Supplement: Supplementary file 5 [file Table5.docx]

**Supplementary Table 5 - Patient and clinical data characteristics**

| *N = 640* | Audit 1  N = 377 | Audit 2  N = 263 | P-value |
| --- | --- | --- | --- |
| Population  *0-16*  *17-65*  *>65* | 70 (18.6%)  **267 (70.8%)** 40 (10.6%) | 57 (21.7%)  **188 (71.5%)**  18 (6.8%) | 0.203 |
| Gender  *Male*  *Female* | **210 (55.7%)**  167 (44.3%) | **169 (64.3%)**  94 (35.7%) | **0.037** |
| Immune status  *General population*  *High risk population* | **344 (91.2%)**  33 (8.7%) | **239 (90.9%)**  24 (9.1%) | 0.87 |
| Reasons for consultation  *Pain*  *Others, including:*   - *Swelling* - *Control* - *Prosthetic problem* - *Dental trauma* | **296 (78.5%)**  81 (21,5%)  *17 (4.5%)*  *11 (2.9%)*  *33 (8.7%) 20 (5.3%)* | **208 (79.1%)**  **55 (20,9%)**  *14 (5.3%)*  *13 (4.9%)*  *8 (3.0%)*  *20 (7.6%)* | 0.862 |
| Diagnosis  Pulpal and periapical diseases *  Others, including:   - Periodontal diseases - Dental trauma - Other oral and dental *infections*** - *Non-urgent problem* | **227 (60.2%)**  150 (39.8 %)  *27 (7.2%)*  *21 (5.6%)*  *46 (12.2%)*  *56 (14.8%)* | **159 (60.5%)**  104 (39.5 %)  *17 (6.5%)*  *23 (8.7%)*  *33 (12.5%)*  *31 (11.8%)* | 0.950 |
| Dental interventions  Performed  Not performed, for reasons:  *Clinical situation*  *Infection context*  *Intervention difficulty*  *Non urgent visits*  *Lack of organization* | **227 (60.2%)**  150 (39.8%)  *23 (15.3%)*  *40 (26.7%)*  *31 (20.7%)*  *22 (14.7%)*  *34 (22.7%)* | **135 (51.3%)**  128 (48.7%)  *29 (22.7%)*  *29 (22.7%)*  *19 (14.8%)*  *33 (25.8%)*  *18 (14.1%)* | **0.026** |

* Caries, acute infections, symptomatic pulpitis, pulpopathy

** *osteitis, cellulitis, tooth eruption* and infectious diseases of the oral mucosa.
