# Supplementary material for: Appropriateness of antibiotic prescribing in dental practice: results of two clinical audits conducted at a university hospital
Source: Front Oral Health. 2026 Sep 18;7:1913184. doi: 10.3389/froh.2026.1913184 (PMC13630595; doi:10.3389/froh.2026.1913184)
Supplement: Supplementary file 6 [file Table6.docx]

**Supplementary Table 6.** Comparison of appropriate (A1) and inappropriate (I2) prescriptions by audit period.

| **Characteristic** | **Audit 1 — A1 (n=31)** | **Audit 1 — I2 (n=93)** | **Audit 2 — A1 (n=22)** | **Audit 2 — I2 (n=47)** |
| --- | --- | --- | --- | --- |
| **Reason for consultation, n (%)** | | | | |
| Pain | 19 (61.3%) | 84 (90.3%) | 17 (77.3%) | 43 (91.5%) |
| Other¹ | 12 (38.7%) | 9 (9.7%) | 5 (22.7%) | 4 (8.5%) |
| **Diagnosis, n (%)** | | | | |
| Pulpal & periapical² | 0 (0.0%) | 71 (76.3%) | 1 (4.5%) | 41 (87.2%) |
| Other diagnoses³ | 31 (100%) | 22 (23.7%) | 21 (95.5%) | 6 (12.8%) |
| **Management strategy, n (%)** | | | | |
| Dental intervention | 7 (22.6%) | 35 (37.6%) | 5 (22.7%) | 19 (40.4%) |
| Antibiotic without intervention | 24 (77.4%) | 58 (62.4%) | 17 (77.3%) | 28 (59.6%) |
| **Reason for antibiotic without intervention (A1: 24/17; I2: 58/28), n (%)** | | | | |
| Clinical situation | 0 (0.0%) | 12 (20.7%) | 0 (0.0%) | 11 (39.3%) |
| Infectious context | 22 (91.7%) | 9 (15.5%) | 17 (100%) | 5 (17.9%) |
| Difficulty performing intervention | 1 (4.2%) | 12 (20.7%) | 0 (0.0%) | 4 (14.3%) |
| Organizational constraints | 1 (4.2%) | 25 (43.1%) | 0 (0.0%) | 8 (28.6%) |

*¹²³ See footnotes to Table 1.*
